# The I2 Test for Selection Bias Risk Assessment in Single Trials: Recommended Simulated Comparator Trial (SCT) Settings

**DOI:** 10.7759/cureus.68911

**Published:** 2024-09-07

**Authors:** Steffen Mickenautsch, Veerasamy Yengopal

**Affiliations:** 1 Faculty of Dentistry, University of the Western Cape, Cape Town, ZAF; 2 Community Dentistry, University of the Witwatersrand, Johannesburg, ZAF

**Keywords:** clinical trial appraisal, i2, review of clinical trials, selection bias, systematic review and meta analysis

## Abstract

Selection bias in clinical trials is a form of systematic error and may be detected using the I² test with a 0/>0% threshold (bias: I² > 0%, no bias: I² = 0%). The test operates on the premise that effective randomisation eliminates in-between study heterogeneity beyond the play of chance in a baseline variable meta-analysis of all the trial’s baseline variables. Since the I² statistic was originally designed to measure in-between study heterogeneity in meta-analyses, the test requires the generation of at least two simulated comparator trials (SCTs). During this process, three parameters are set: SCT sample size (N_SCT_), the minimum-maximum range of random values (R_SCT_), and the number of generated SCTs to be used (SCT_N_). Each of these parameters influences the 0/>0% threshold of the resulting I² point estimate, thereby affecting the test’s sensitivity in indicating a positive result. The purpose of this technical report is to highlight the effect that SCT parameters have on the test’s accuracy and to recommend appropriate parameter settings.

## Introduction

Selection bias occurs when patients in a clinical trial are not randomly allocated to treatment groups, leading to one group having characteristics that may falsely make its treatment appear more effective. It is a form of systematic error based on knowledge about patient characteristics conducive to successful trial outcomes and knowledge about the allocation of such patients in a specific sequence of test and control interventions. If such knowledge is used to allocate these patients exclusively to a particular test intervention, a more favourable outcome over the control intervention can be achieved [1].

The risk of selection bias in clinical trials is appraised during systematic reviews using tools such as the Cochrane risk-of-bias 2 tool, which are currently based on qualitative text analysis of trial reports [2]. Such methods scan the text of a trial report for possible indicators of selection bias but are limited by the possibility that certain facts about trial conduct may not be included. In contrast, quantitative methods that analyse reported trial data for bias would provide a more objective risk appraisal. One quantitative method is the Berger-Exner test, but it has the disadvantage of relying on individual patient data, which are seldom reported in the supplementary material of trial reports [3]. Another commonly used method is the formal testing of baseline differences for statistical significance (p < 0.05). However, significant baseline differences can occur by chance [4], and non-significant results may not ensure the absence of selection bias [5].

Mickenautsch and Yengopal (2024) proposed another possible test for selection bias risk appraisal in clinical trials [6]. The test relies on the reported data of baseline variables per intervention group and uses the I² point estimate with a 0/>0% threshold to determine whether selection bias risk is present (I² > 0%) or not (I² = 0%). The I² point estimate is typically used to evaluate the consistency of results across multiple studies, but in this case, it has been adapted to assess whether the randomisation in a single trial was successful. The rationale of the test is based on the premise that effective randomisation will result in a lack of between-study heterogeneity beyond the play of chance, reflected as 0% I² in a baseline variable meta-analysis for all baseline variables [7, 8].

Because the I² statistic was designed for measuring between-study heterogeneity in meta-analyses, the test requires the generation of at least two ‘simulated comparator trials (SCTs)’ before the analysis can be undertaken. SCTs consist of simulated baseline data with artificially set between-study homogeneity to create a ‘perfect world’ scenario where no selection bias exists. This lack of heterogeneity is reflected by an I² = 0% value in a fixed-effect meta-analysis of SCTs. A clinical trial is tested for selection bias risk by adding its baseline data to the SCT meta-analysis. If the resulting I² point estimate is also 0%, the test result is considered negative, indicating no selection bias risk for the tested trial. If the I² is greater than 0%, the test result is considered positive, suggesting high selection bias risk [6].

The purpose of this technical report is to highlight the effect that SCT parameters have on the test’s accuracy and to recommend appropriate parameter settings.

This manuscript has been published online as a preprint in Authorea: Mickenautsch S, Yengopal V. The I^2^ -test for selection bias risk assessment in single trials: recommended simulated comparator trial (SCT) settings (Preprint). Authorea. 2024, 10.22541/au.172322841.14013846/v1.

## Technical report

SCTs were generated by creating three parallel data columns in MS Excel (Microsoft Corporation, Redmond, Washington) [6]: an ascending list of integers (1, 2, 3, …) serving as data point IDs (Column 1), a random allocation sequence for two groups, A and B (Column 2), and a list of randomly selected values (integers or decimals with random duplications allowed) within a specified range (minimum-maximum value) sorted in ascending order (Column 3). To conduct the selection bias test, a minimum of two SCTs were generated, each with its own individual random allocation sequence and random values.

**Figure 1:**
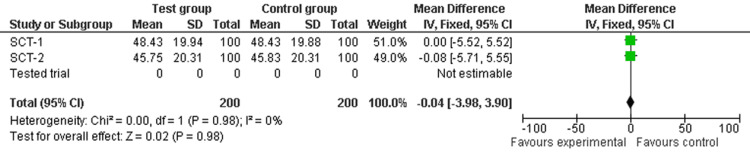
Meta-analysis of simulated comparator trials (SCT)

**Figure 2:**
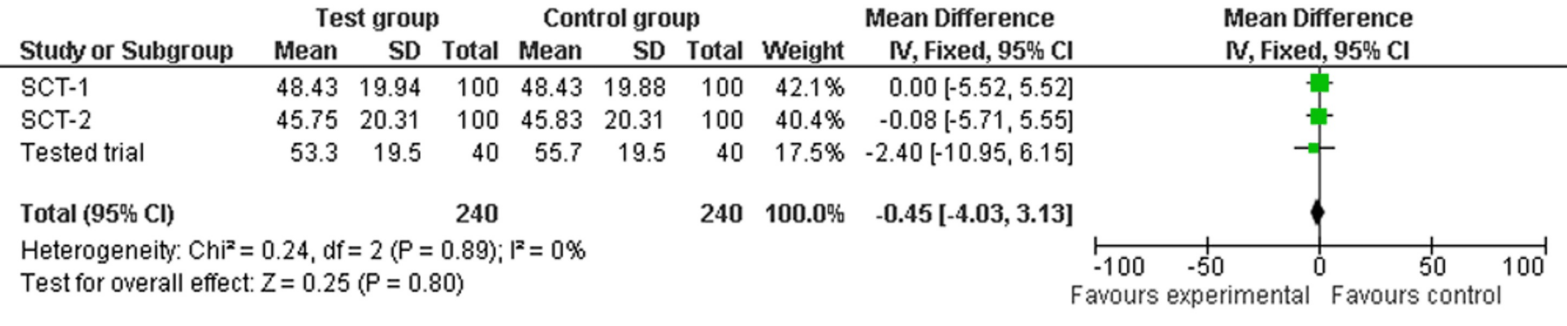
Trial test for selection bias risk

The test was performed by: (i) sorting the random values in Column 3 according to the allocation to Groups A and B in Column 2 in MS Excel and calculating the mean value (with standard deviation (SD)) for Groups A and B per SCT, (ii) entering the mean (SD) with the sample size (N_SCT_) per group for each SCT into a fixed-effect meta-analysis (using Review Manager - RevMan 5.0.24 software, London, United Kingdom) and confirming the resulting 0% I² point estimate, as shown in Figure 1. This figure, generated by us, demonstrates that sorting SCT random values according to a random A-B sequence produced an absolute random allocation of values to Groups A and B, without any differences between the two groups beyond the play of chance. Next, (iii) the mean (SD) value and sample sizes for Groups A and B from the trial being tested were added to the same meta-analysis, and (iv) the analysis was repeated, recording the new I² point estimate, as shown in Figure 2.

If the new I² point estimate was also 0%, the test result was considered negative, and no selection bias risk for the tested trial was assumed. If the point estimate was I² > 0%, the test result was considered positive, and the tested trial was assumed to have a high selection bias risk [6].

During the test, three parameters were set: the total number of data points (SCT sample size, N_SCT_), the minimum-maximum range of random values (R_SCT_) (the same values set for each SCT), and the number of generated SCTs used in the meta-analysis (SCT_N_). Each of these parameters affects the 0/>0% threshold of the resulting I² point estimate and, thus, the test’s sensitivity to indicate a positive result (I² > 0%). These threshold changes can be demonstrated by stepwise increasing the difference between the mean values of Groups A and B in a simulated test trial with the initial settings: sample mean, x̄₁ = 1.00, SD₁ = 1.00, N₁ = 1 (Group A); x̄₂ = 1.00, SD₂ = 1.00, N₂ = 1 (Group B) (Appendices-Section 1.1). The higher the N_SCT_ and the lower the R_SCT_ and S_CTN_, the lower the 0/>0% threshold of the I² point estimate and, therefore, the more sensitive the test becomes.

**Figure 3:**
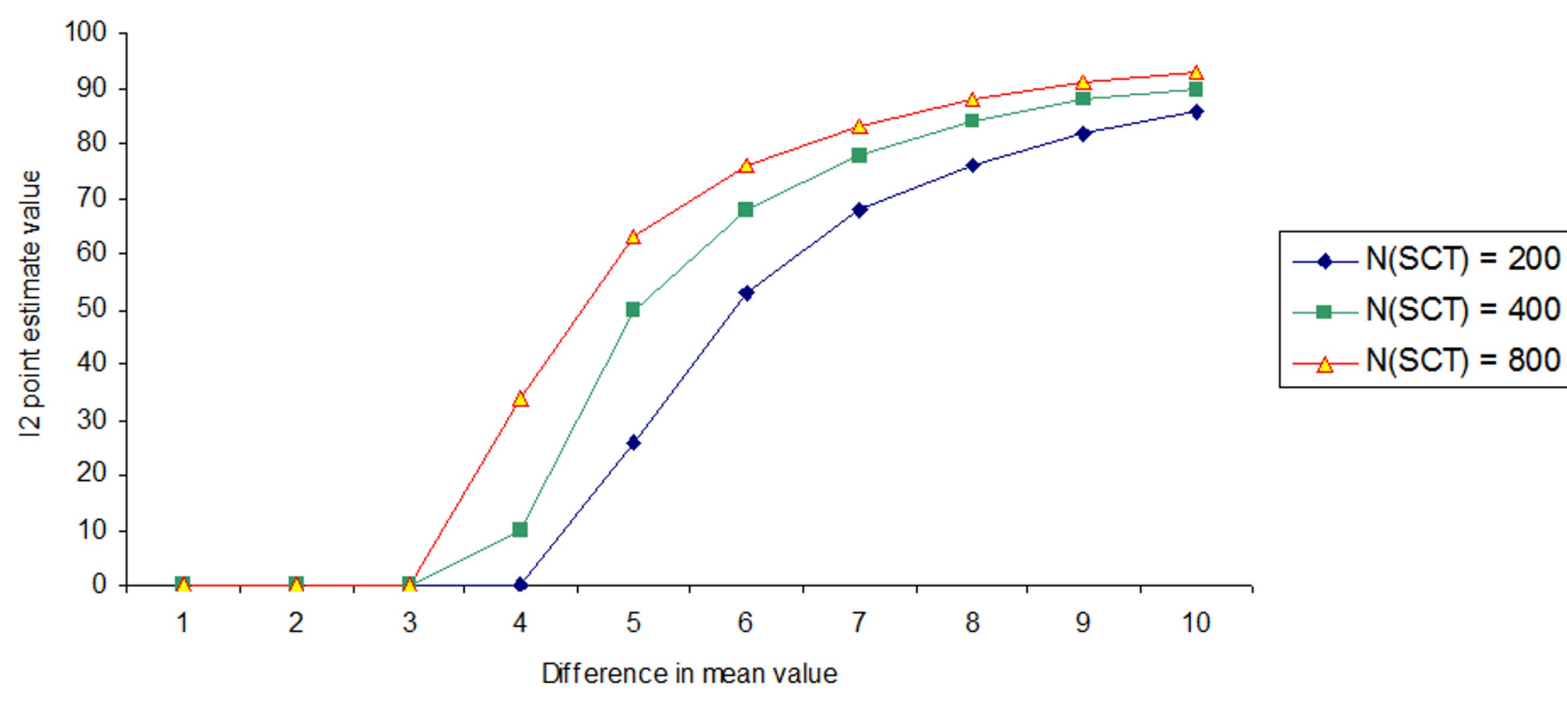
Differences in the I2 point estimate due to different SCT sample sizes (NSCT)

For example, when both SCTs were set at N_SCT_ = 200, the I² point estimate indicated significant selection bias (I² > 0%) at a mean difference of 4.00. However, when both SCTs were set at N_SCT_ = 800, the I² point estimate indicated significant selection bias already at a mean difference of 3.00 between Groups A and B (Figure 3 and Appendices - Section 1.2).

**Figure 4:**
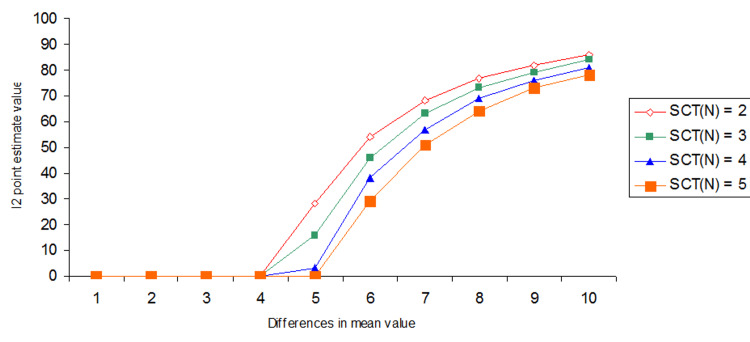
Differences in the I2 point estimate due to different SCT number (SCTN)

A test with two SCTs indicated significant selection bias (I² > 0%) at a mean difference of 4.00, while a test with five SCTs indicated significant selection bias only at a mean difference of 5.00 between Groups A and B (Figure 4 and Appendices-Section 1.3).

**Figure 5:**
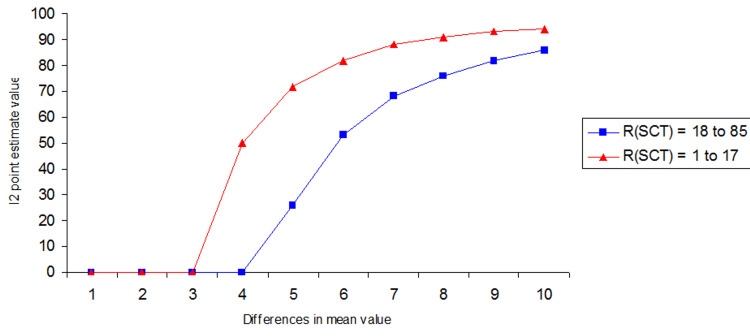
Differences in the I2 point estimate due to different range (RSCT)

A range (R_SCT_) of 16 (Min/Max = 1-17) indicated significant selection bias (I^2^ > 0%) at a mean difference of 3.00, while an R_SCT_ of 67 (Min/Max = 18-85) indicated significant selection bias only at a mean difference of 4.00 between Groups A and B (Figure 5 and Appendices-Section 1.4).

Hence, the setting of appropriate test parameters (N_SCT_, R_SCT_, and SCT_N_) is important to assure test reliability. We recommend limiting the number of SCTs to SCT_N_ = 2 and setting the N_SCT_ according to that of the baseline variable, as reported in the trial to be tested. If the trial sample size is less than N = 100 per treatment group, the N_SCT_ should be set at 200 (i.e., 100 for Groups A and B per SCT). The R_SCT_ minimum and maximum values should be set according to the mean (SD) value of the baseline variable from both groups combined. The steps to be followed for such an estimation are presented in Appendices-Section 2.

## Discussion

In this technical report, we have demonstrated that the I^2^ point estimate varies with the number of SCTs included in the baseline variable meta-analysis, the SCT sample size, and the specified range of variable values. Such observed variability sets limits to the estimate’s purpose as a reliable tool for testing selection bias risk in single trials and is in line with previous observations. 

Rücker et al. established that the I^2^ point estimate increases with the number of subjects included in trials, based on the observation that the I^2^ point estimate tended to be 100% by artificially inflating the sample size under a random effects model meta-analysis [9]. In addition, von Hippel noted that a too-small trial number (N = 7) in a meta-analysis causes an overestimation of the actual in-between trial heterogeneity reflected by an artificially increased I^2^ point estimate [10].

Mickenautsch and Yengopal [11] established that trial number and sample size in a baseline variable meta-analysis did not affect the test accuracy of the I^2^ point estimate when the cut-off point was set at 0% or when selection bias was completely absent. However, it was also noted that in simulated cases where the presence of selection bias was artificially assured, the mean (SD) I^2^ point estimates were larger, with 95.66 (0.85)% and 98.98 (0.13)% for meta-analyses, with only five trials, compared to 92.89 (1.3)% and 98.02 (0.13)% for meta-analyses with 15 trials. In addition, in line with Rücker et al. [9], larger I^2^ point estimates were observed when the trial sample sizes were larger [11]. From these observations, it can be concluded that the setting of sample size, variable range and trial number for SCT generation cannot be arbitrary but needs to follow evidence-based considerations in order to avoid systematic reviewer error during selection bias risk appraisal.

We recommend setting the SCT sample size and variable range (N_SCT_ and R_SCT_) in line with the values reported for the test trial to assure reviewer independence from these parameter values. Because the I^2^ point estimate is smaller with smaller SCT sample sizes and wider variable range, the I² = 0%/I² > 0% threshold is higher and thus raises the danger of a false negative appraisal result. In that way, high selection bias risk (I^2^ > 0%) may intentionally be masked for a trial during its appraisal in a systematic review if these parameters are set intentionally too lenient. Setting the SCT sample size and variable range in line with that of the test trial will also assure that the trial will be tested in the baseline variable meta-analysis against comparators that, albeit highly ideal in terms of in-between study heterogeneity, may still not be too dissimilar.

A systematic review of meta-epidemiological studies established that trials with smaller sample sizes (N < 100) have statistically significantly larger effect estimates in comparison to trials with at least 100 subjects per intervention group (ratio of odds ratios (ROR) 0.67; 95% CI: 0.54-0.82) [12]. The observed overestimation of 33% of the true treatment effect associated with smaller trials may be ascribed to systematic error (bias), which may be more prevalent in this type of smaller study. Hence, if trials with smaller sample sizes are appraised by the use of the I^2^ test, SCTs with higher sample sizes should be used. Setting this higher SCT sample size for smaller trials at N_SCT_ = 200 (i.e., 100 per Groups A and B) will lower the I^2^ = 0/I^2 ^> 0% threshold in line with meta-epidemiological findings [12] and increase the sensitivity of the test for correctly identifying the higher selection bias risk in small trials.

We further recommend limiting the number of SCTs to two, which is the minimum required trial number for conducting a meta-analysis. It is correct that too small a trial number in an outcome meta-analysis of clinical trials may cause an overestimation of the actual in-between trial heterogeneity [10]. However, such overestimation should not affect a 0% I^2^ point estimate in a baseline variable meta-analysis when true random allocation to trial intervention groups has been effective and thus the only possible source of heterogeneity, a difference in baseline values beyond the play of chance, is absent [7]. Because the I^2^ describes the percentage of total variation across studies that is due to heterogeneity and not due to chance [8], mere chance baseline differences in the presence of too small a trial number should not raise the point estimate above 0%.

## Conclusions

The I^2^ test for selection bias risk assessment in single trials involves the use of SCTs. The parameters of these SCTs (N_SCT_, R_SCT_, and SCT_N_) affect the I^2^ = 0/I^2 ^> 0% threshold: the higher the N_SCT_ and the lower the R_SCT_ and SCT_N_, the lower the threshold. If the threshold is set too high, trials with a high selection bias risk may not be correctly identified. In this technical report, we provided justification for setting the parameters at the following levels: SCT_N_ = 2 and N_SCT_ and R_SCT_ in accordance with the baseline values reported in the test trial, provided the test trial had a sample size of at least N = 100 for each intervention group. For smaller trials, the sample size of all SCTs should be set at N_SCT_ = 200 (100 for Groups A and B each).

## Appendices

All appendices and data are made fully available without restriction and can be freely downloaded from https://data.mendeley.com/datasets/w873kczfgw/1.
